# Reliability of T-WSI to Evaluate Neighborhoods Walkability and Its Changes over Time

**DOI:** 10.3390/ijerph17217709

**Published:** 2020-10-22

**Authors:** Daniela D’Alessandro, Diego Valeri, Letizia Appolloni

**Affiliations:** Department of Civil, Building and Environmental Engineering, Sapienza University of Rome, 00184 Rome, Italy; diego.valeri@uniroma1.it (D.V.); letizia.appolloni@uniroma1.it (L.A.)

**Keywords:** walkable neighborhoods, physical activity, healthy urban planning, reliability, reproducibility

## Abstract

More walkable neighborhoods are linked to increased physical activity. The Walking Suitability Index of the territory (T-WSI) is an easy method to evaluate walkability on the basis of direct observation. T-WSI provides 12 indicators divided into 4 categories (practicability, safety, urbanity, pleasantness); the weighted analysis of these indicators gives an overall score of the actual usability of the neighborhood. The aim of the study is to evaluate the ability of T-WSI’ indicators to measure, in a reliable way, any street’s walkability variations occurred over time. The investigation was performed in 2018 in nine urban neighborhoods of Rieti city. Cronbach’s α is used to evaluate internal consistency of T-WSI; Intraclass Correlation Coefficient (ICC) is used to evaluate the reproducibility of measurements (or ratings) made by different investigators. Cronbach’s α is 0.89 (± 0.02); ICC is also good (ICC = 0.89; 95% CI: 0.84–0.92). The results of the 2018 investigation are also compared with those collected in 2016 in the same districts. The results show that T-WSI is a reliable and easy to use tool, useful to measure the effectiveness of the interventions already realized at local level, but it could also contribute to making decisions to develop regeneration projects.

## 1. Introduction

According to the European Environment Agency (EEA), around a quarter of the European Union’s territory is dedicated to urban uses and cities tend to expand with increasing demand for construction; currently more than 50% of the population lives in cities and it is estimated that this percentage will increase to reach 70% by 2050 [1,2].

In general, urbanization and the cities’ structure offer numerous opportunities for health, providing availability and easier accessibility to health services [3].

An effective strategy for European cities which has potential for health and well-being is focused on the reduction of a sedentary lifestyle. Physical activity is today considered one of the best investments for health and sustainable development and it is one of the sub-objectives of the United Nations Sustainable Development Goals (SDGs) that must be achieved by 2030 [4]. It has been documented that if all types of physical activity increased, this would help obtain health and economic benefits thanks to the reduction of incidence and complications of most chronic diseases [5,6,7].

The WHO Action Plan for Physical Activity [8] has set the reduction of a 10% sedentary status by 2025 and of a 15% by 2030 and proposes it to be based on a systemic approach. This implies the implementation of a collective and coordinated response among all the interested factors that contribute to define the contexts in which people live, work and play to guarantee a more active future. Basically, the action plan aims to guide governments to promote physical activity and health through the creation of active societies and living environments, with a view to sustainability in its comprehensive meaning.

The conceptual models, which support research on the built environment and the inhabitants’ behavior, show how much the physical context influences human activities [9]. Apparently, the subjective nature of the built environment does not allow one to easily mark out the principles of design of the places that can be transferred to any context [9]. To better understand the relationship between living environment, human behavior and health, it is necessary to underline the complexity and the interactivity of the relationship between context and individuals [10].

Frequently, in the scientific literature, a compact city with high density was considered healthier than urban forms characterized by scattered settlements and low residential density; in fact, the presence of a land-use mix, the frequent road intersections between residential and commercial areas, etc., facilitate direct pedestrian paths between the various destinations [11].

The widespread city, with a low density, defined in relation to health as an Obesogenic Urban Form [11], is in general characterized by residential areas, commercial units and offices far from one another, a fact that requires daily travel, mostly by private transportation. Several studies defined a dynamic relationship between the physical characteristics of the compact city and the ability to perform physical activity in urban spaces.

For example, Sallis et al. (2016) observed a direct relationship between physical activity and residential density, the density of the intersections, the density of public transport and the number of parks and green spaces within a radius of one km from the house [12]. Moreover, a longitudinal study [13] carried out in Australia on 11,000 adults (40–65 years) from 200 districts of Brisbane estimated the effects of changes in the characteristics of the neighborhood that occurred over a period of six years on the tendency to walk of the residents; the increase in residential density, functional mix and road connectivity was associated with a modest but significant increase in the average walk duration.

Also in Australia, the RESIDential Enviroments (RESIDE) [14] study reports the results of an active planning policy implemented by the Government (called ”Livable Neighborhoods”) in which, in light of previous studies, some performance requirements of the districts were deemed to be able to determine an increase of pedestrian movements. These requirements were applied, in a different design combination of 73 new suburban residential settlements, which were classified in four sub-groups of neighborhoods. In this study, the authors reported the results obtained six years after the change of residence. The best results in terms of active transport recourse were observed in the neighborhoods where an adequate functional mix of public transport, proximity to various destinations and easy access to all the infrastructures has been achieved. However, the interesting aspect concerns the quantification of the effect, which in these districts has led to a 2.64 times higher probability of walking than taking vehicle transportation, a probability that has significantly remained higher even for more than a one hour walk.

Obviously, the direct relationship between the compact city and increase of physical activity varies depending on the age group. In a systematic review of studies aimed at assessing the relationship between aspects of the built environment and moderate–vigorous physical activity of young people (including walking) [15], the physical characteristics of the compact city (residential density, density of intersections, traffic lights, crossings, etc.) have been associated with a reduction in daily physical activity for children aged 8–9 years. In children and adolescents, the daily activity showed an increase (8–16%) in neighborhoods with green spaces or space to play.

In fact, proximity to parks and recreational environments is generally associated with more frequent physical exercise and a more active lifestyle [16].

Takano et al. (2002), analyzing the five-year residence of 3144 elders, have observed that residents of neighborhoods equipped with spaces for walking, parks and tree-lined roads lived significantly longer than the others [17]. In fact, the availability of green spaces in the housing context plays an important health role, helping to mitigate climate impacts, to improve the environmental quality and the psychological wellbeing of the population, to promote social cohesion and to reduce inequalities [18].

Although many studies support the role of green space as a promoter of physical activity and well-being, it is, however, important to highlight that several aspects, not yet well clarified, make it difficult to use the information obtained from the literature for design purposes; the evidence derives mainly from observational studies, where the cause–effect relationship cannot be evaluated. Furthermore, there aren’t yet single definitions of green spaces (parks, cycle paths, gardens, etc.) compared to the effectiveness detected, nor are reliable indications regarding the optimal distance of the available green space from the house and regarding the best dimensions to promote physical activity more than scarcely available [16].

However, green areas are not always available in neighborhoods, especially in historic and consolidated cities. In these cases, if the goal is to make the population walk, especially the sedentary part of it, it is important to adapt the neighborhoods to the new needs: for example, by making some areas walkable, ensuring the usability of the roads within them, improving the environmental quality, optimizing public transport and encouraging citizens’ participation. The literature is rich in experiences that document the effectiveness of good practices adopted by many municipalities in this direction [19].

A critical aspect in the decision-making process is the measurement of the built environment impact on the walkability. In general, three approaches are used [20,21]: (a) self-reported interviews and questionnaires (self-reported measures); (b) objective observations (audit), performed by expert personnel; (c) measures deriving from archive datasets, often reworked with GIS (Geographic Information Systems). The major limitations of these approaches depend, for the most part, on the difficulty of finding reliable data to describe reality, from the time required to collect information, to the size of the survey areas taken as a reference [22].

In an attempt to overcome these limitations, an instrument called the “index of territorial suitability for walking” has been developed (T-WSI: Walking Suitability Index of the Territory) [21,22,23]. Its purpose is to measure the usability of an area on foot, which coincides with what today is defined as an “environmental island”, i.e., a circumscribed area, generally bounded by axes of the main road network, in which the residential function prevails: it is a part of the city affected by special rules of circulation that limit excessive speeds. Basically, it is the ideal setting for getting around on foot.

As already described elsewhere [21,22,23], T-WSI does not follow origin–destination logic, because the interest is not focused on a precise path. The main objective of the tool is to understand how the territory in question is walkable as a whole, to identify any shortcomings to be remedied; this is in order to allow all users to move freely on foot in the neighborhood, to carry out daily activities. In fact, the length of the journeys to be traveled to reach activities and services can influence a more or less dynamic attitude [24,25]. Equally important are the characteristics of the routes, both in terms of actual practicability (presence of obstacles, road surface, slope) and of safety in its widest meaning [21].

This study aims to evaluate the ability of T-WSI to measure these indicators and to detect, in a reliable way, any variations of their values occurring over time. Therefore, two investigations performed over time in the same environmental areas by different observers have been compared. The observers applied the same methodology.

## 2. Materials and Methods

### 2.1. Study Area

Rieti is a small city of 47,912 inhabitants, located in Latium Region (Central Italy). It is a typical provincial Italian town, with a car access-restricted city center; in the inner city, walking is still the main modal option for performing everyday errands.

Around the city center, a series of neighborhoods were developed mostly during the first part of the 20th century. As described in a previous paper [23], differences between the city center and the surrounding areas are numerous: (i) the former, a self-standing city developed for non-motorized modes; the latter, satellite-conceived, urbanized to progressively meet the motorization requirements; (ii) mixed land use in the city center vs the dominant residential use of the more modern districts; (iii) the hilly morphology of the historic city versus the flatter areas of the 20th century development.

**Figure 1:**
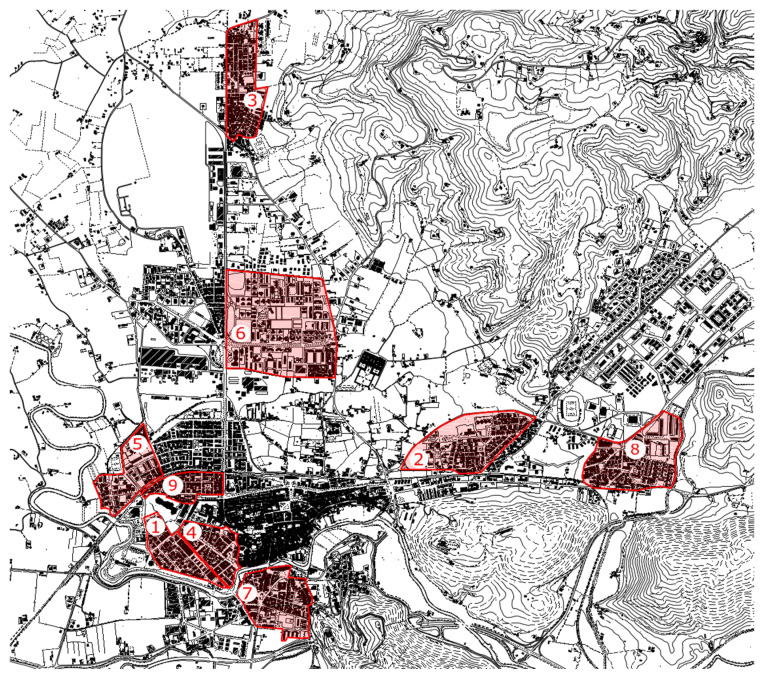
Rieti map. The areas in red are those included in the study. **1.** Città Giardino, **2.** Piazza Tevere, **3.** Quattro Strade, **4.** Fiume dei Nobili, **5.** Molino della Salce, **6.** Micioccoli, **7.** Borgo S. Antonio, **8.** Villa Reatina, **9.** Viale dei Flavi.

The target area of the study includes nine districts out of a total of 15 (60%), accounting for about 55% of the built surface of the town (Figure 1); in these districts, about 33% of the population lives. The inner city was not included in the investigated area, since, by nature, it is already “pedestrian-friendly”. In fact, the study focused on other districts, where, due to their characteristics, the motorization process competes with walking.

The districts’ selection was based on two criteria: (1) the possibility to compare neighborhoods with different building characteristics; (2) the real possibility to promote interventions to improve the roads’ usability for walking into the neighborhoods.

In order to measure the districts’ level of walkability, the investigation areas had a 300–400 m radius, corresponding to about a 5 min walking distance. This size matches to these of “environmental areas”, as defined elsewhere [21] and to sizes suggested by several authors in the literature [26,27,28,29].

### 2.2. Data Collection and Data Analysis

In order to measure walkability, T-WSI was used [21,22,23]. This index measures the walkability level of a district, taking into account the five Ds of urban forms (Density, Diversity, Design, Destination accessibility, and Distance to transit), because they are considered important factors affecting walking behaviors in neighborhoods [29,30]. The T-WSI tool is an audit based on direct observation by a trained investigator. As described in a previous article [23], unlike the usual methods to assess walkability, T-WSI is not designed to evaluate the walking-friendliness of a given origin–destination paths or trips, but of the whole walking environment where such activities take place, independently from the distance to walk.

The T-WSI characteristics, methods of use and evaluation criteria have been already described in previous studies [21,22,23]. In summary, it provides 12 indicators divided into 4 categories (practicability, safety, urbanity, pleasantness). The weighted analysis of these indicators gives an overall score of the actual usability of the neighborhood [23]. A different weight is assigned to each category and to the included indicators, considering its impact on walkability; the weight of each indicator has been defined by a multidisciplinary panel of experts. A value is assigned to each indicator according to a pre-arranged scale of merit, corresponding to a numerical rating scale (excellent = 1, good = 0.7, poor = 0.35, bad = 0.0). Data collected in each street are inserted in an algorithm to perform weighted sums and to aggregate indicators and categories (C), according to percentiles and decimal coefficients, up to defining the final index (final street index = 0.30 × C—Practicability + 0.25 × C—Safety + 0.22 × C—Urbanity + 0.23 × C—Pleasantness). The neighborhood index results from the sum of the weighted averages of each street index, in which the length of each street is considered [21,22,23].

To assess the tool’s ability to consistently measure what it is supposed to measure, the statistical analysis was divided into two phases: the first aimed at verifying the reliability and internal consistency of the measuring instrument; the second aimed at testing the coherence or reproducibility of the quantitative measurements made by different investigators on the same neighborhoods.

To test internal consistency, Cronbach’s α was estimated [31,32]. Internal consistency refers to how closely a set of walkability-related neighborhood factors is related as a group, which indicates the consistency of the scales in reflecting the construct they measure. Cronbach’s alpha of 0.70 and higher was set as an acceptable value for reliability scales [33]. As already described in a previous study [23], in this investigation Cronbach’s alpha test results reported α-values always above the requested 0.70 threshold, with an overall value of 0.890 (± 0.02), showing the T-WSI reliability in measuring walkability.

To assess the reproducibility of the data collection performed by different observers, the Intraclass Correlation Coefficient (ICC) was used. According to Mokkink et al. [34], reliability is defined as the extent to which scores for indicators which have not changed are the same for repeated measurement under varying conditions. In practice, the ICC is a measure of the reliability of two different raters to measure the subjects similarly [35]. The ICC is a widely used index for reliability assessment, such as test–retest, intra–rater and interrater reliability analyses [29]. Interrater reliability demonstrates how much a tool is robust to changes in raters; thus, a scale with high interrater reliability is less prone to measurement error (e.g., variation in human judgement) [35]. Therefore, the ICC is used to determine the consistency of measurements (reliability): a higher ICC indicates greater consistency [36].

In particular, in this study the objective was to compare the measurements performed by different surveyors in evaluating the same quantity (interrater reliability) [37]. Therefore, in 2018, two trained surveyors collected the audit data in the same environmental areas in two independent investigations (test–retest). To each surveyor, a detailed card containing the interpretation criteria for each parameter was provided, in order to support their decision in the answer’s choice. In total, the statistical evaluation regarded the comparison of 173 streets distributed in 9 out of 15 neighborhoods of Rieti city.

**Figure 2:**
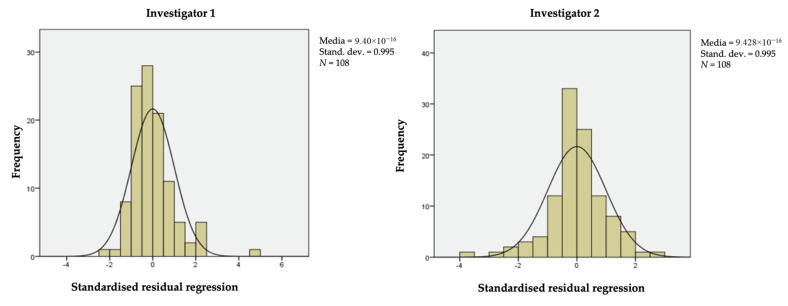
Standardized residual regression of data obtained in the investigations.

The ICC used for this study is that applied for a two-way random-effects ANOVA system [35,38], since the data collected by the first and the second investigator are modeled as random effects. With the random variables *π_I_* (raters) and *λ_j_* (encounters) assumed to be independent and distributed with *N*(0,$\sigma_{2}^{\pi }$), *N*(0,$\sigma_{2}^{\lambda }$) (see the standardized residuals regressions reported in Figure 2), the ICC formula (Equation (1)) for the model is:

(1) $$\mathrm{ICC}=\frac{\sigma_{2}^{\lambda }}{\sigma_{2}^{\lambda }\;+\sigma^{2}}$$

where *σ_λ_* is the variance between encounters, and *σ_π_* is the variance of the raters within encounters.

The ICCs results were interpreted following ratings suggested by Landis and Koch [39]: 1.0–0.8 (almost perfect), 0.8–0.6 (substantial agreement), 0.6–0.4 (moderate agreement), 0.4–0.2 (fair agreement), and 0.2–0.0 (poor agreement).

In order to evaluate changes in the T-WSI indexes occurring in the same districts over time, two data collections performed in 2016 and in 2018 were compared. A *T*-test for independent samples was used to evaluate the significance of the differences between indexes calculated in the two investigations.

To calculate reliability tests and *T*-tests, Excelpackage was used.

## 3. Results

### 3.1. Reliability of T-WSI

The overall ICC value, calculated comparing the results of both data collections, shows a very good reproducibility level (ICC = 0.89; 95% CI: 0.84–0.92) and a high coherence among the observers. The results then reveal a high level of measurement concordance between the data independently collected by investigators.

In light of the observed reliability, the results of two investigations performed in 2016 and 2018 have been compared, in order to detect variations in T-WSI scores within districts during time.

### 3.2. Investigated Neighborhoods’ Characteristics

The study was performed in nine neighborhoods (precisely, those numbered in the Figure 1), involving about 55% of the built surface of the city, in which about 63% of the population live staying outside the inner city (15,469 inhabitants—inh).

Table 1 shows the neighborhoods’ characteristics. They markedly differ in terms of size and population density: the average density of the whole study area is about 8304 inh/km^2^, ranging from Molino della Salce, the less densely inhabited (5962.3 inh/km^2^), to Città Giardino, the most inhabited (11,052.3 inh/km^2^).

**Figure 3:**
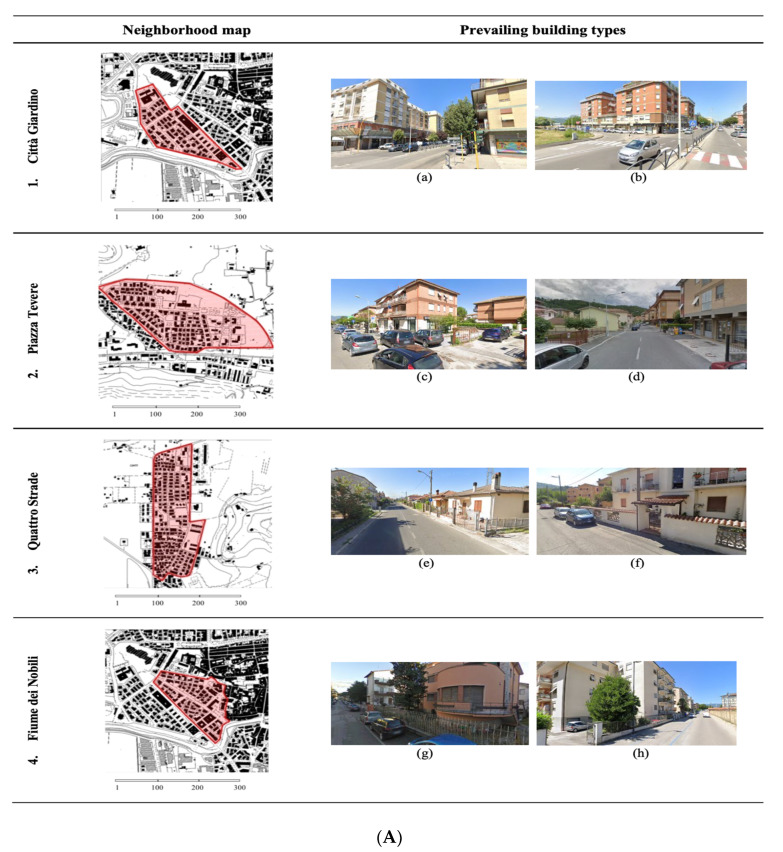

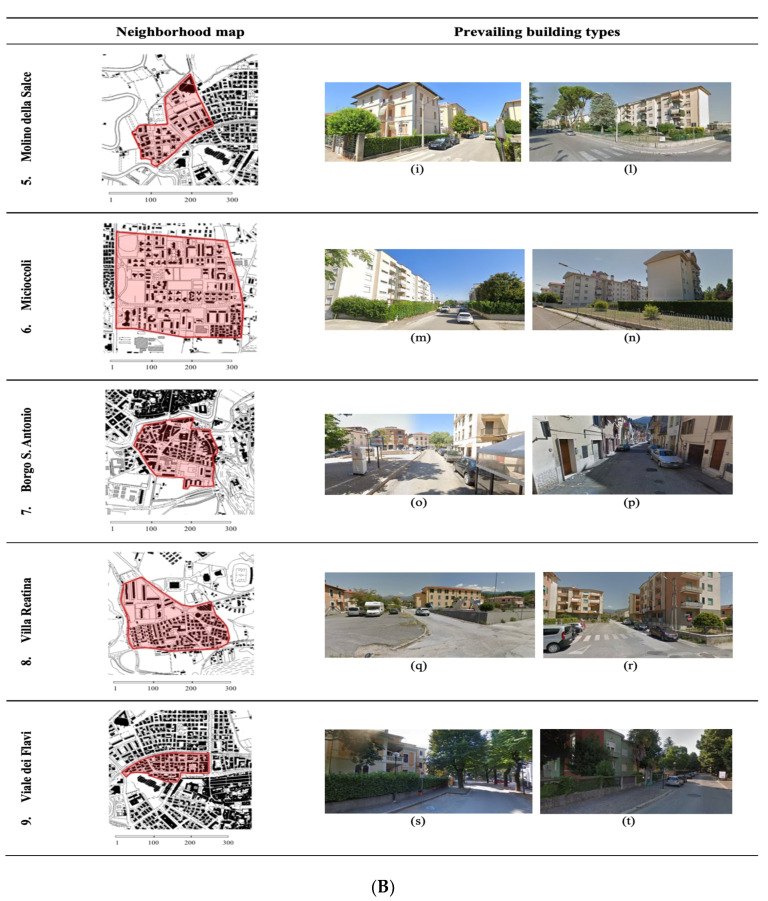
(**A**) Neighborhood map and prevailing building types of Città Giardino (1—a,b), Piazza Tevere (2—c,d), Quattro Strade (3—e,f), Fiume dei Nobili (4—g,h) districts. (**B**) Neighborhood map and prevailing building types of Molino della Salce (5—i,l), Micioccoli (6—m,n), Borgo S. Antonio (7—o,p), Villa Reatina (8—q,r), Viale dei Flavi (9—s,t) districts.

Figure 3A,B shows map and prevailing building types of each investigated neighborhood.

As already discussed in previous studies [22,23], the observed density differences depend on these neighborhoods including various building typologies and belonging to different historical periods. The Borgo S. Antonio neighborhood (n.7 in Figure 3B—letters o,p), partially included in the central historical city, is a mainly residential area characterized by in-line, block, open courtyard buildings, terraced buildings and small villas, mostly built between 1950s and 1960s [22,23].

The same building consistency characterizes the districts of Città Giardino (n. 1 in Figure 3A—letters a,b), Fiume dei Nobili (n. 4 in Figure 3A—letters g,h), and Molino della Salce (n. 5 in Figure 3B—letters i,l), which are called ”middle-class neighborhoods” and were built starting from the middle of the ‘50s of the last century. All three are located west of the historic center.

The neighborhoods of Villa Reatina, defined as the “working-class” “district” (n. 8 in Figure 3B—letters q,r), and of Quattro Strade (n. 3 in Figure 3A—letters e,f), are mainly residential, low population density, characterized in part by single-family buildings built between the 1940s and 1965s and partly from 2–3 storey buildings (mostly of economic-popular extraction) built between the 1960s and 1990s [22,23].

The districts of Micioccoli (n. 6 in Figure 3B—letters m,n) and Piazza Tevere (n. 2 in Figure 3A—letters c,d), considered periurban residential areas, date back to the historical period between the 1960s and 1990s and have been brought to completion in our time. They are neighborhoods with a defined building type, with a prevalence of in-line, block, and terraced buildings [22,23].

The neighborhood called Viale dei Flavi (n. 9 in Figure 3B—letters s,t), built mainly in the fascist period (1920s–1940s) and completed in the 1950s, includes public buildings, little villas, courtyard houses, and blocks of flats. This area is located near the historic center and is characterized by high urban quality [22,23].

### 3.3. Neighborhood Difference in T-WSI between 2016 and 2018 Investigations

Table 2 and Table 3 show the T-WSI indexes collected in the two investigations performed in the same districts in 2016 and in 2018. In four districts (Piazza Tevere, Quattro Stade, Micioccoli, and Viale dei Flavi), the T-WSI obtained a slightly lower score in 2018 than in 2016, while it improved in the other three districts (Fiume dei Nobili, Borgo S. Antonio, and Villa Reatina).

In general, the Safety and Urbanity categories show the lower scores in both investigations.

Major intra-district differences in the T-WSI index value between the two investigations are observed in three neighborhoods: Borgo S. Antonio (from 36.79 to 51.55), Villa Reatina (from 24.21 to 36.95), and Viale dei Flavi (from 61.16 to 52.12); Borgo S. Antonio alone shows a significant difference (*p* = 0.0097) (Table 4). The reason for these differences could be mainly attributed to maintenance works performed (or not performed) in the city between 2016 and 2018. This hypothesis is confirmed by the fact that the principal variation in scores regards the indicators included into practicability, which is significantly improved in Micioccoli (from 58.1 to 76.9; *p* = 0.0280) and Borgo S. Antonio (from 40.3 to 75.5; *p* = 0.0006). In other neighborhoods (Piazza Tevere and Viale dei Flavi), between the first and the second surveys, the conditions of the infrastructures worsened, but the difference is not significant. This could be due to poor maintenance of the sidewalks and the presence of numerous fixed and temporary obstacles along the routes.

The significant difference in the practicability index observed in the Micioccoli neighborhood (from 58.1 to 76.9; *p* = 0.0280) depends on the maintenance works undertaken precisely regarding the renovation and maintenance of the sidewalks and the removal of architectural barriers. In particular, it should be noted that for the Villa Reatina and Micioccoli districts there are two neighborhood’ contracts which, among the various interventions, also contain projects aimed at increasing and improving infrastructures (pedestrian and cycle paths) that encourage active mobility. At the same time, Micioccoli shows a significant worsening in security (from 52.2 to 31.4; *p* < 0.0001) and urbanity scores (from 51.2 to 33.0; *p* = 0.0318) in the two-year period. This partly depends on the presence of a large degraded area of social housing in the neighborhood, which has not been affected by redevelopment programs.

Furthermore, also in Piazza Tevere, the safety category shows worsening results in the second investigation (from 48.3 to 24.1; *p* = 0.0002), in particular for the inadequacy of artificial lighting and the absence of protection from vehicles.

A highly significant improvement in urbanity index is instead observed in the Quattro strade district (from 7.2 to 16.7; *p* < 0.0001), although the score continues to be low, mainly for the inadequacy of the sidewalks and the roads equipment.

Finally, the pleasantness index shows a significant improvement in the Villa Reatina district (from 54.1 to 72.5; *p* < 0.0033) only, thanks to a reduction in vehicular traffic, an improvement in the building context and in the green spaces.

## 4. Discussions and Conclusions

Measuring the walkability of the different streets with suitable and reliable instruments is fundamental. The ICC is an instrument used in several research fields to measures the reliability of two different raters to evaluate a subject similarly [29,35] and, in this case, the subject is the walkability of a district.

In particular, the assessment of the interrater reliability of instruments is essential when proposing novel evaluation tools, as with the T-WSI [35]. Although the ICC is subject to a variety of methodological issues [35,40,41], it is widely applied in the research in order to measure reliability.

In this study, the ICC shows a high level of concordance between raters and this is an important result, as it demonstrates that the evaluation tool is robust to changes in raters, and hence is less prone to measurement error.

Therefore, the statistical analysis carried out showed that the T-WSI is a reliable, efficient, and reproducible tool. As said before [23], T-WSI is an effective tool to assess where people really can walk and orient transport planning towards decisions where walking can be considered one strategic criterion. T-WSI can also provide useful directions about the ease of reaching local facilities by people living in the study area or nearby (e.g., post office, pharmacy, etc.). By assessing which could be the more comfortable, safer or attractive link, in relation to its length, it is possible to analyze where it is easier for people to walk, in terms of street appeal or perceived safety and so on.

The comparison between the measures performed in 2016 and 2018, allowed us to verify the T-WSI’s ability to detect even small differences, too, as it was observed in the Borgo S. Antonio district and for some categories, also, in Micioccoli, Molino della Salce, Piazza Tevere and Villa Reatina.

The literature suggests that the characteristics of the neighborhoods influencing walking include the presence and quality of the pedestrian and bicycle paths, but also the presence of a functional mix, the proximity and accessibility to various destinations and the presence of greenery, as well as an acceptable level of traffic [12,13,14,16,17,18,19,24,25]. In the investigated environmental areas of Rieti city several of these characteristics are unsatisfactory: pedestrian infrastructures are lacking or poorly designed; if present, they highlight problems especially in terms of dimensions and maintenance of the sidewalk surface.

The scores regarding the presence of a functional mix on the streets were insufficient in all the investigated environmental areas. This does not indicate the absence of commercial or tertiary activities but highlights their irregular distribution on the territory. In fact, in the examined neighborhoods the commercial activities and offices serving all districts are generally concentrated in one or two streets or in central areas. This distribution does not interfere with the proximity and accessibility of these activities, since the neighborhoods are small and the distances are limited, but it certainly makes the streets lacking in commercial activities less attractive and uncrowded and, consequently, the streets are perceived as less safe. Furthermore, safety perception is worse due to the frequent lack of artificial lighting dedicated to pedestrians.

Considering that the literature shows a strong relationship between safety perception and the population’s propensity to walk [42,43,44,45], this problem should be the subject of particular attention both in the definition of urban plans for sustainable mobility (PUMS), and in the regeneration interventions in the suburbs of cities, areas most often affected by these problems.

Other factors considered essential to increasing physical activity are the presence of green spaces and poor traffic flows [16,17,18,19,25,30]. These factors were measured with the T-WSI and the scores were good in almost all environmental areas for both indicators. It should be highlighted, however, that although there are no high levels of traffic in the neighborhoods, it would be necessary to improve the usability of the areas and the safety of pedestrians by inserting elements to moderate the speed of the vehicles (bollards, 30 areas, 30 limited traffic zones, etc.), not present in the environmental areas studied.

At the same time, introducing vegetation into the courtyards of buildings or in the immediate vicinity provides spaces useful for relaxing, contributing to producing a greater sense of belonging and to reducing crime rates [8,18,43]. Numerous studies, indeed, have documented how the level of exposure to natural environments affects health and the physical activity level, in addition to having an important role in mitigating the impacts of the built environment on the urban climate [17,18,43]. In the light of this study’s results, it could be of particular relevance for the Fiume dei Nobili district, where a serious lack of greenery has been observed.

Overall, the lacks found in the investigations could be resolved with simple and low-cost interventions. Furthermore, the study offers several suggestions in order to carry out urban redevelopment and/or regeneration projects. These interventions represent a great opportunity, if properly managed, to reduce inequalities and respond to the health and economic problems of cities [43,46,47,48,49,50]. At the same time, the COVID-19 pandemic has highlighted the need to understand if and how the people’s lifestyle in a neighborhood will be modified. In this context, it is crucial to plan and program the neighborhood functional mix, taking into account some essential urban social functions such as living, working, supplying goods and services, providing care and assistance, learning, and enjoying recreational spaces [50].

In light of what has been described, the existence of a dynamic relationship between the built environment and health [9] is evident. Having an easy-to-use, valid, and reliable tool to evaluate the characteristics of the territory and its ability to attract people for walking can facilitate the decision-making processes [51,52], and support and direct the choices concerning the priorities of interventions and investments in designing neighborhoods for people [53].

Obviously, policies, actions and projects must be able to adapt to the continuous transformations that characterize our era, from climate change, to economic uncertainty, to housing crises [46,54], to an aging population, on to pandemics, and to transfer such information and factors into the representation of the places.

As required by the UN SDGs [55], built environments and urban spaces should be flexible and resilient enough to take on all the changes that affect them, both those in progress and those in future that are today difficult to predict [45].

## Figures and Tables

**Table 1:** Neighborhood characteristics.

Neighborhood	Street	Surface	Population	Population Density
	(No)	(m^2^)	(No)	(Inhabitants/km^2^)
1. Città Giardino	17	124,408	1375	11,052.3
2. Piazza Tevere	20	273,879	1765	6444.5
3. Quattro strade	21	198,866	2138	10,750.9
4. Fiume dei Nobili	16	119,400	824	6901.2
5. Molino della Salce	18	153,462	915	5962.3
6. Micioccoli	27	571,395	3562	6233.9
7. Borgo S. Antonio	25	210,383	1849	8788.7
8. Villa Reatina	19	282,320	2303	8157.4
9. Viale dei Flavi	10	91,820	738	8037.4
Total	173	2,025,933	15,469	7635.5

**Table 2:** Average results of indicators, indexes and the Walking Suitability Index of the territory (T-WSI) obtained in each neighborhood in the investigation performed in 2016.

Neighborhood	Practicability (P)				Safety (S)				Urbanity (U)				Pleasentness (PL)				T-WSI Index
	Sidewalk Surface	Obstacles	Road Slope	Practicability Index	Protection from Veichles	Road Lighting	Crossing Protection	Safety Index	Sidewalk Width	Road Equipment	Land Use Mix	Urbanity Index	Vehicular Traffic	Building Context	Green Spaces	Pleasentness Index	
1.Città Giardino	66	71	98	78.6	13	27	47	30.2	41	28	37	36.1	63	73	54	63.3	53.6
2.Piazza Tevere	85	92	91	88.8	31	40	69	48.3	24	16	42	28.5	68	88	34	63.9	59.7
3.Quattro strade	18	13	85	40.5	0	30	9	12.5	5	0	14	7.2	77	82	23	61.6	31.0
4.Fiume dei nobili	37	37	100	59.2	2	42	44	30.3	29	0	44	23.7	45	69	9	41.1	40.0
5.Molino della Salce	76	65	91	78.4	47	31	31	35.8	32	8	56	34.1	57	56	25	46.8	50.4
6.Micioccoli	46	60	71	58.1	45	72	42	52.2	62	54	37	51.2	53	65	57	58.3	55.2
7.Borgo S. Antonio	29	21	67	40.3	12	59	21	29.9	8	14	44	22.3	62	71	26	53.6	36.8
8.Villa Reatina	14	14	23	16.9	2	16	6	8.0	16	12	34	21.4	72	56	31	54.1	24.2
9.Viale dei flavi	86	76	97	87.6	8	47	39	31.9	57	46	31	45.1	81	90	49	73.8	61.2

**Table 3:** Average results of indicators, indexes and T-WSI obtained in each neighborhood in the investigation performed in 2018.

Neighborhood	Practicability (P)				Safety (S)				Urbanity (U)				Pleasentness (PL)				T-WSI Index
	Sidewalk Surface	Obstacles	Road Slope	Practicability Index	Protection from Veichles	Road Lighting	Crossing Protection	Safety Index	Sidewalk Width	Road Equipment	Land Use Mix	Urbanity Index	Vehicular Traffic	Building Context	Green Spaces	Pleasentness Index	
1.Città Giardino	75	71	79	75.3	0	44	47	31.3	35	40	54	42.9	61	60	50	57.3	53.1
2.Piazza Tevere	76	68	81	75.9	0	37	33	24.1	21	24	41	28.9	87	74	48	70.9	51.5
3.Quattro strade	24	22	28	24.8	0	30	9	12.5	8	12	30	16.7	77	83	29	63.8	28.9
4.Fiume dei nobili	69	58	64	64.7	0	48	61	38.0	17	14	48	27.1	44	76	17	45.5	45.3
5.Molino della Salce	91	70	91	85.7	0	30	51	28.8	14	24	41	25.7	27	81	54	52.0	50.5
6.Micioccoli	70	70	90	76.9	10	46	37	31.4	30	37	33	33.0	70	69	52	64.0	52.9
7.Borgo S. Antonio	69	58	96	75.5	0	32	46	27.1	27	26	51	35.1	68	87	31	62.3	51.5
8.Villa Reatina	41	34	47	41.2	4	19	9	10.9	22	8	37	23.6	94	83	35	72.5	36.9
9.Viale dei flavi	76	67	87	77.4	0	47	26	24.4	39	27	40	36.2	70	79	44	64.5	52.1

**Table 4:** Comparison and significance differences among indexes and the Walking Suitability Index of the territory (T-WSIs) obtained in each neighborhood in 2016 and 2018 investigations.

Neighborhood	Practicability (P)			Safety (S)			Urbanity (U)			Pleasentness (PL)			T-WSI		
	2016	2018	*p*-Value	2016	2016	*p*-Value	2016	2018	*p*-Value	2016	2018	*p*-Value	2016	2018	*p*-Value
1.Città Giardino	78.6	75.3	ns	30.2	31.3	ns	36.1	42.9	ns	63.3	57.3	ns	53.6	53.1	ns
2.Piazza Tevere	88.8	75.9	ns	48.3	24.1	0.0002	28.5	28.9	ns	63.9	70.9	ns	59.7	51.5	ns
3.Quattro strade	40.5	24.8	ns	12.5	12.5	ns	7.2	16.7	<0.0001	61.6	63.8	ns	31.0	28.9	ns
4.Fiume dei nobili	59.2	64.7	ns	303	38.0	ns	23.7	27.1	ns	41.1	45.5	ns	40.0	45.3	ns
5.Molino della Salce	78.4	85.7	ns	35.8	28.8	0.0267	34.1	25.7	ns	46.8	52.0	ns	50.4	50.5	ns
6.Micioccoli	58.1	76.9	0.0280	52.2	31.4	<0.0001	51.2	33.0	0.0138	58.3	64.0	ns	55.2	52.9	ns
7.Borgo S. Antonio	40.3	75.5	0.0006	29.9	27.1	ns	22.3	35.1	ns	53.6	62.3	ns	36.8	52.5	0.0097
8.Villa Reatina	16.9	41.2	ns	8.0	10.9	ns	21.4	23.6	ns	54.1	72.5	0.0033	24.2	36.9	ns
9.Viale dei Flavi	87.6	77.4	ns	31.9	24.4	ns	45.1	36.2	ns	73.8	64.5	ns	61.2	52.1	ns

ns = not significant.
